# A Study on the Biofilm Removal Efficacy of a Bioelectric Toothbrush

**DOI:** 10.3390/bioengineering10101184

**Published:** 2023-10-13

**Authors:** Hyun Mok Park, Seungjae Ryu, Eunah Jo, Sun Kook Yoo, Young Wook Kim

**Affiliations:** 1 PAIST (ProxiHealthcare Advanced Institute for Science and Technology), Seoul 04513, Republic of Korea; hmpark@proxihealthcare.com (H.M.P.); sjryu@proxihealthcare.com (S.R.); eajo@proxihealthcare.com (E.J.); 2 Graduate Program of Biomedical Engineering, Yonsei University, Seoul 03722, Republic of Korea; 3 Department of Medical Engineering, Yonsei University College of Medicine, Seoul 03722, Republic of Korea

**Keywords:** bioelectric effect, microcurrent, toothbrush, dental, biofilm, plaque

## Abstract

Effective oral care is a critical requirement to maintain a high quality of life. Most oral diseases are caused by plaque (oral biofilm), which is also correlated with systemic diseases. A common method to remove biofilm is brushing teeth with toothpaste. However, 3.5 billion people in the world have oral diseases, meaning that more efficient methods of removing biofilms are needed. We have developed a toothbrush that applies a bioelectric effect (BE) utilizing an electric force for biofilm removal. It demonstrated significantly higher biofilm removal efficiency than non-BE manual toothbrushes. Tests were performed in saline and toothpaste conditions using various pressures. Results showed that the BE toothbrush had a significantly higher biofilm removal efficiency in saline (0.5 N: 215.43 ± 89.92%, 2.5 N: 116.77 ± 47.02%) and in a toothpaste slurry (0.5 N: 104.96 ± 98.93%, 2.5 N: 96.23 ± 35.16%) than non-BE manual toothbrushes. Results also showed that BE toothbrushes were less dependent on toothpaste. This study suggests that the application of BE can be a new solution to plaque problems in oral care.

## 1. Introduction

To enhance quality of life, maintaining optimal oral health is paramount. Regrettably, according to the World Health Organization (WHO), approximately 3.5 billion individuals globally are afflicted by oral diseases [1,2]. These oral diseases (including periodontitis, gingivitis, etc.) arise due to the inadequate management of biofilms, commonly referred to as plaque [3,4]. Plaque is characterized as a deposit forming a biofilm composed of adherent bacteria within the oral cavity [4]. This biofilm can trigger inflammation in periodontal tissues. It has been identified as a significant contributor to gingivitis and periodontitis [5]. Notably, deposits of plaque within the oral cavity can manifest clinical indications within 10 days [6,7]. Hence, to avert or manage periodontal diseases, it becomes imperative to either remove or impede the formation of plaque [8]. Efficient plaque removal proves pivotal in regulating periodontal diseases.

Notably, plaque has direct correlations with systemic illnesses such as stroke [9], diabetes [10], cardiovascular disease [11], and Alzheimer’s disease [12]. Consequently, the effective removal of plaque is important for both public and private healthcare. Plaque is typically removed through a mechanical method using a toothbrush or oral products [13,14]. In the 1960s, electric toothbrushes that increased plaque removal efficiency were developed [15].

Nevertheless, with 3.5 billion people worldwide suffering from oral diseases, typical plaque removal methods might not be effective. Therefore, a more efficient means of removing plaque is needed.

Toothpaste is commonly employed during tooth-brushing routines. While toothpaste could effectively aid in plaque removal, ongoing discussions regarding its safety arise due to the presence of abrasives, surfactants, and fluoride [16,17,18]. Overdosing with fluoride has been associated with adverse effects such as skeletal deformities, cancer, and gastric mucosa damage [18,19,20]. Among pregnant women, certain studies have indicated potential risks of birth defects [21]. Additionally, concerns are being raised, particularly concerning children, regarding potential serious issues such as teeth and skeletal underdevelopment [17,22,23].

Plaque is a type of biofilm that forms in the oral cavity [4]. The durability of a biofilm extends to the point where antibiotic resistance becomes 500 to 5000 times more pronounced than that exhibited by individual bacteria [8,24,25].

Studies on photodynamic therapy to remove biofilm have been reported [26,27,28]. Photodynamic therapy (PDT) is a proposed method to treat oral diseases and plaque by applying a combination of light, photosensitizers, and oxygen [26,27,28]. However, PDT has disadvantages in terms of time and pain [28].

Biofilms consist of polysaccharides and electrically polarized bacterial cells [29,30]. External electrical stimuli can disrupt biofilms’ structural integrity and impact the metabolic state of bacteria [28,29,30,31]. As demonstrated in a recent study, the combined application of an electrical signal known as the bioelectric effect (BE) along with both direct current (DC) and alternating current (AC) is remarkably effective for biofilm removal [31,32,33,34,35,36,37].

Recently, our group developed a toothbrush with a bioelectric effect and demonstrated its effectiveness in reducing plaque-induced dental gingival index in clinical trials [8,35]. This result means that applying a bioelectric effect to oral products has a clinically significant effect on oral care.

However, clinical trials have the disadvantage of being difficult to quantitatively evaluate due to variations among subjects [38]. In vitro tests are more convenient and more reproducible than in vivo tests. Therefore, they are widely used in research related to the human body [38,39,40]. Likewise, in relation to toothbrushes, studies using various in vitro brushing simulators have been reported [41,42,43,44].

In this study, we quantitatively compared the plaque removal efficiency of a bioelectric toothbrush and a regular toothbrush to observe the bioelectrical effect on plaque. We performed a quantitative plaque removal efficiency test using a developed brushing simulator for four types of toothbrushes (two types of regular toothbrushes and the proposed toothbrush (with or without BE)). The experiment was conducted under two conditions (with only saline and with toothpaste slurry) to investigate whether BE could replace toothpaste. Based on experimental results, we investigated the plaque removal efficiency of BE and toothpaste dependence for each.

## 2. Materials and Methods

### 2.1. Tested Toothbrushes and Design of BE Toothbrush

**Figure 1:**
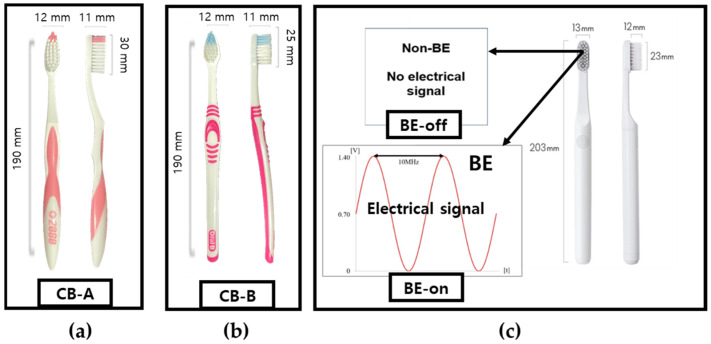
Actual figure of tested toothbrushes: (**a**) CB-A, (**b**) CB-B. (**c**) Proposed toothbrush (applied non-BE: BE-off, applied BE: BE-on).

The tested toothbrushes and their abbreviations are listed in Table 1 and Figure 1. We aimed to compare the plaque removal efficiency of a toothbrush with BE. Two commonly used toothbrushes and two developed toothbrushes were tested under two conditions: BE off and on. These conditions were specified to investigate the effect of the bristles (typical toothbrush versus proposed toothbrush) on plaque removal.

BE applies a combination of electrical signals to remove biofilms. The electrical signal was a combination of a 0.7 V sinusoidal signal at a frequency of 10 MHz with a DC offset of 0.7 V, as shown in Table 2 [8,45].

**Figure 2:**
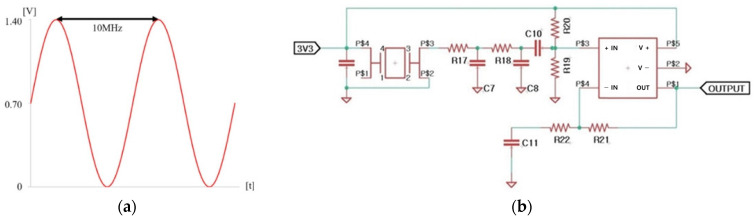
(**a**) Example of bioelectric effect signal; (**b**) schematic of electronic system for generating the bioelectric effect signal [8,45].

The frequency of the electrical signal was selected based on previous studies. The DC offset was set below the electrolysis threshold of 0.82 V [31]. Our previous study revealed that this signal does not induce electrolysis [45]. The electrical signal is shown in Figure 2a. An electronic circuit for generating the output signal was developed, as shown in Figure 2b. Electronic circuits were embedded in the proposed toothbrush. A stainless-steel electrode was chosen due to its corrosion resistance and conductivity essential for supplying an electric field. The signal for BE is output through these two electrodes.

The current from the signal output was measured using a multimeter (EDU34450A, Keysight Technologies, Santa Rosa, CA, USA) in a 0.9% saline condition. The measured current was about 40.7 ± 1.5 μA (*n* = 3). The current of BE was determined to be safe because it was within the biocompatible current range [46].

### 2.2. Development of Brushing Simulator

**Figure 3:**
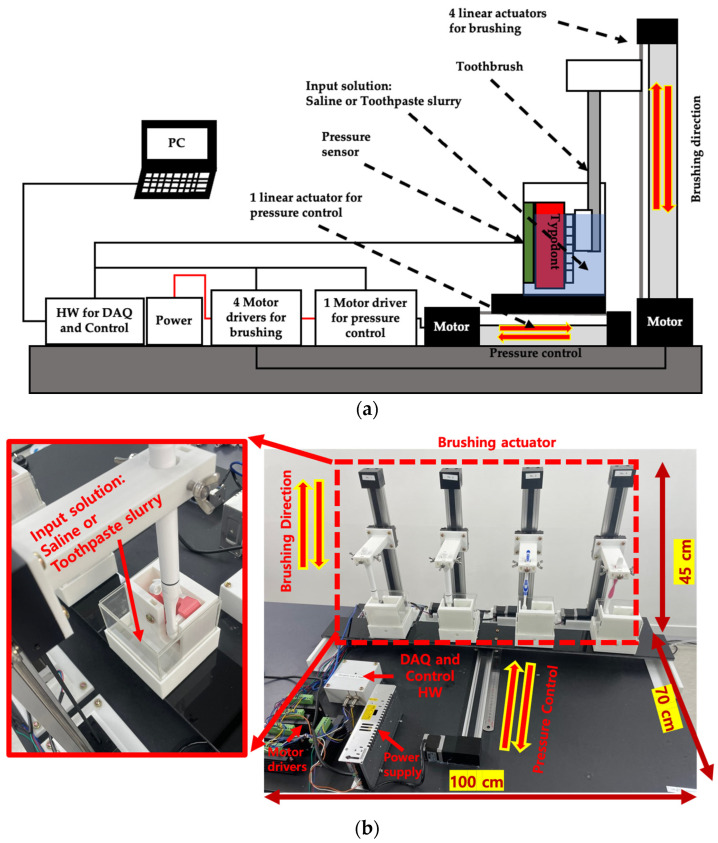
The development of the brushing simulator: (**a**) Schematic of the brushing simulator; (**b**) actual figure of the brushing simulator.

The brushing simulator consists of five linear actuators with a stepper motor (Scipia, Gwangju, Republic of Korea), five motor drivers (TB6600, DFRobot, Shanghai, China), four piezoelectric pressure sensors (QA3040P, Marveldex Inc., Bucheon, Republic of Korea), a switching-mode power supply (HU10748-13002E, Yueqing Hengwei Electric Co., Yueqing, China), and a construct of typodonts and toothbrushes. The construct was produced using a 3D printer (X1-carbon, Bambu lab, Shenzhen, China) and polylactic acid (PLA). A standard typodonts was used to construct the brushing simulator. For the brushing control and pressure exerted by the toothbrush data acquisition (DAQ), we used an Arduino Uno (Arduino.cc, Ivrea, Italy). The toothbrushing simulator was capable of simulating toothbrushing at up to 150 strokes/min in a 5 cm driving range. The brushing simulator is shown in Figure 3.

**Figure 4:**
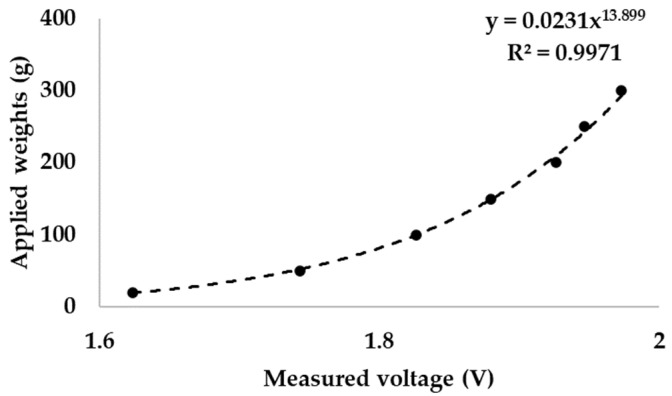
Calibration of the piezoelectric pressure sensor corresponding to a previous report [47].

The pressure sensor of the simulator was calibrated by applying various masses from 20 to 300 g. The result showed a standard correlation of the piezoelectric material (Figure 4). A piezoelectric pressure sensor can be calibrated using a power function [47]. A standard weight was applied to the pressure sensor and the output voltage was recorded (*n* = 3). According to ISO/TR 14569-1, the pressure that the toothbrush presses on an artificial tooth is between 0.5 and 2.5 N [48]. The measured weight was converted to newtons (N).

### 2.3. Plaque Culture

*Streptococcus mutans* (KCTC 3065, Korean Collection for Type Cultures (KCTC), Jeongeup, Republic of Korea) used in this research were distributed from KCTC. They were cultured in a growth medium (LB Broth, Ambrothia Inc., Daejeon, Republic of Korea) at 37 °C for 48 h to provide sufficient time for maturation.

### 2.4. Experiment of Plaque Removal

**Figure 5:**
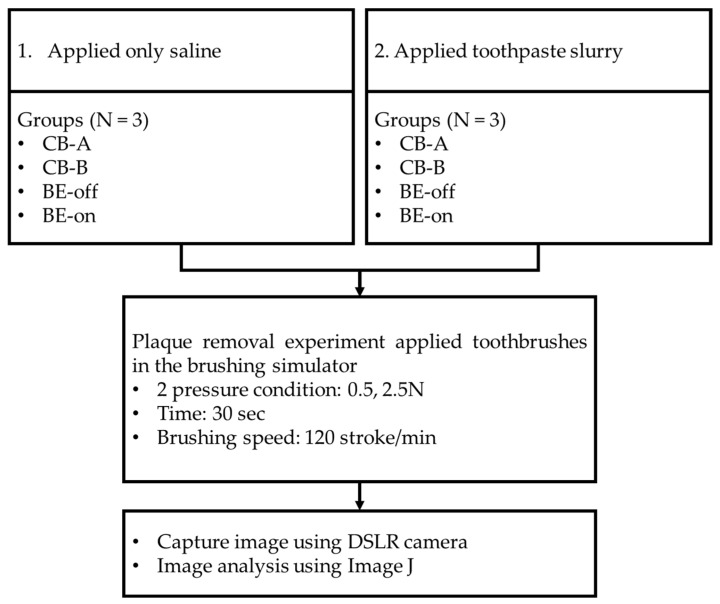
Graphic description of the plaque removal procedure.

Typodonts were evenly coated with the cultured *S. mutans*. Green marker spray (OccludeTM, Pascal, Bellevue, WA, USA) was used to visualize *S. mutans.* (plaques) [40]. The coated typodont was mounted on the brushing simulator. The force that the toothbrush pressed on the tooth was set to be 0.5 N and 2.5 N. According to ISO/TR 14569-1, the force that a toothbrush presses on teeth is between 0.5 N and 2.5 N [48]. The molar area was brushed [43]. The brushing frequency was 120 strokes/min [48] for 30 s. The recommended time for brushing is generally 2 min [49,50]. The target area was approximately 1/4 of the entire tooth. The driving range was set to 4 cm as an approximate range of the molar area. After brushing, tested typodonts were eluted in tap water for 30 s. This process was used to remove the plaque that was separated by brushing the teeth so that only the residual plaque would be quantified. According to our preliminary test, it was determined that this process did not cause unintended plaque elution. Plaques of typodonts before and after the experiment were captured under controlled lighting conditions using a DSLR camera (A350, Sony Co., Tokyo, Japan). Image analysis was performed using ImageJ (National Institutes of Health, Bethesda, MD, USA). Two experiments were conducted (*n* = 3). The experimental design is shown in Figure 5.

1. Only saline was applied.
2. A toothpaste slurry (toothpaste–saline at a ratio of 1:3) was applied.

The first experiment (only saline applied) was conducted to determine the biofilm removal effect of using only BE without toothpaste. Saline solution has been used in several studies to simulate saliva [51,52]. The second experiment (toothpaste slurry applied) was designed to represent typical brushing conditions. The toothpaste used was a toothpaste containing fluorine at 1450 ppm (Grate regular-flavor toothpaste, Colgate, Hamilton, NY, USA).

In our previous work, we found that a toothbrush with BE in a clinical trial could significantly reduce the gingivitis index [8,45]. Therefore, an additional comparison was made between a typical toothbrush using toothpaste and a BE toothbrush without toothpaste.

### 2.5. Statistical Analysis

Residual plaques are presented as means and standard deviations (SDs). A two-way analysis of variance (ANOVA) followed by Dunnett’s post hoc test was used to determine the significance of differences between the experimental condition and the CB-A at 0.5 N as control. Paired *t*-tests were used to compare differences between BE-on saline and toothpaste slurry experiments. All statistical analyses were performed using R-studio version 4.3.1 (Posit, Boston, MA, USA). *p*-values of less than 0.05 were considered significant.

## 3. Results

### 3.1. Experiment Using Only Saline

**Figure 6:**
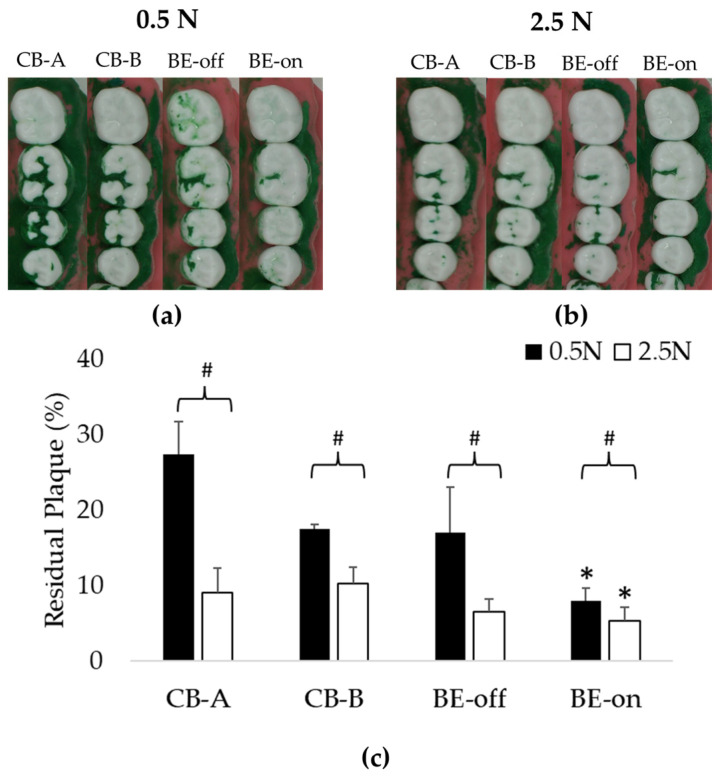
Residual plaques in only-saline experiment: (**a**) Representative image of 0.5 N condition showing significant plaque reduction under BE-on condition; (**b**) representative image of 2.5 N condition showing significant plaque reduction under BE-on condition; (**c**) results of residual plaque using only saline. Results are presented as means ± SDs. * *p* < 0.05 versus CB-A, # *p* < 0.05 versus 0.5 N.

This experiment was designed to evaluate plaque removal efficiencies to observe the effect of BE without using toothpaste. In all pressure conditions, the residual plaque using the BE-on toothbrush was significantly lower than that using other toothbrushes. Toothbrushes except for BE-on showed no significant difference in residual plaque, as shown in Figure 6. A previous study reported that there is no significant difference in plaque removal rate according to the shape of bristles [53]. This result suggests that the high plaque removal rate might be due to the application of BE. The order of residual plaque was 0.5 N > 2.5 N. The higher the pressure with which the toothbrush presses against the teeth, the higher the plaque removal rate. These results are consistent with those of previous reports [54].

At 0.5 N, BE-on showed 7.96 ± 1.67% of residual plaque, while CB-A, CB-B, and BE-off showed 27.42 ± 4.30%, 17.45 ± 0.59%, and 17.03 ± 5.99% of residual plaque, respectively. At 2.5 N, BE-on showed 5.24 ± 1.86% of residual plaque, while CB-A, CB-B, and BE-off showed 9.08 ± 3.18%, 10.20 ± 2.18%, and 6.55 ± 1.59% of residual plaque, respectively. The highest plaque removal efficiency was observed with BE-on at 2.5 N. On average, the plaque removal rate with BE-on was 215.43 ± 89.92% higher at 0.5 N and 116.77 ± 47.02% higher at 2.5 N than those using other toothbrushes.

### 3.2. Experiment Appling a Toothpaste Slurry

**Figure 7:**
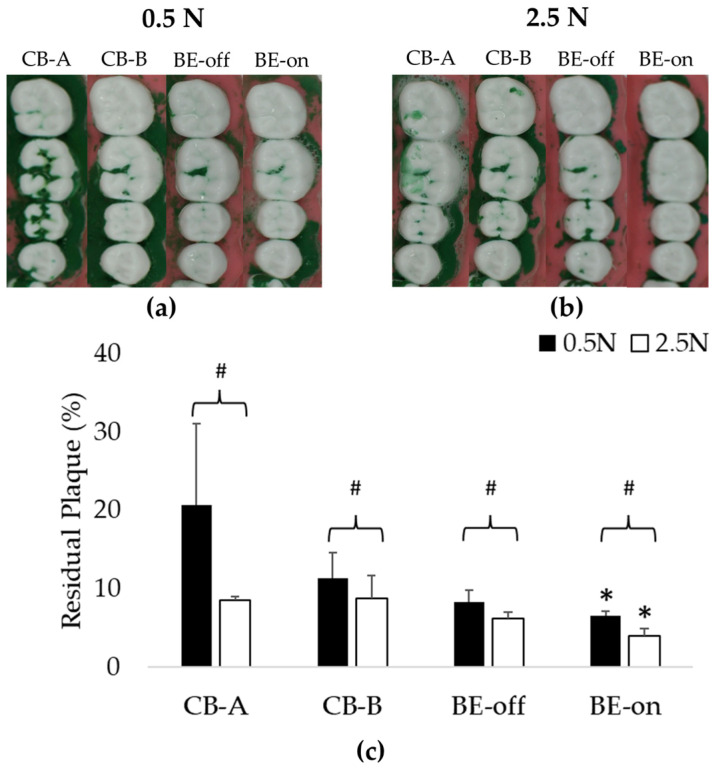
Residual plaques in toothpaste slurry experiment: (**a**) Representative image of 0.5 N condition showing significant plaque reduction using BE-on; (**b**) representative image of 2.5 N condition showing significant plaque reduction using BE-on; (**c**) results of residual plaque after applying a toothpaste slurry. Results are presented as means ± SDs. * *p* < 0.05 versus CB-A, # *p* < 0.05 versus 0.5 N.

This experiment was designed to evaluate plaque removal efficiencies in typical situations. As observed in the experiment applying only saline, the residual plaque using BE-on was significantly lower than in those using other toothbrushes under all pressure conditions, as shown in Figure 7. Using toothpaste can increase plaque removal efficiency [16,17,18]. Therefore, the residual plaque in this experiment using a toothpaste slurry was lower than that when using only saline.

The order of residual plaque was 0.5 N > 2.5 N, as shown in Figure 7. At 0.5 N, BE-on showed 6.54 ± 0.59% of residual plaque, while CB-A, CB-B, and BE-off showed 20.66 ± 10.38%, 11.32 ± 3.24%, and 8.23 ± 1.54% of residual plaque, respectively. At 2.5 N, BE-on showed 3.97 ± 0.97% of residual plaque, while CB-A, CB-B, and BE-off showed 8.51 ± 0.53%, 8.69 ± 2.95%, and 6.19 ± 0.84% of residual plaque, respectively. BE-on was the most efficient in all pressure conditions. The highest plaque removal efficiency was observed for BE-on at 2.5 N. On average, the plaque removal rate using BE-on was 104.96 ± 98.93% higher at 0.5 N and 96.23% ± 35.16% higher at 2.5 N than when using other toothbrushes.

### 3.3. Comparison of Residual Plaque in Applied Saline with BE versus Toothpaste Slurry without BE

**Figure 8:**
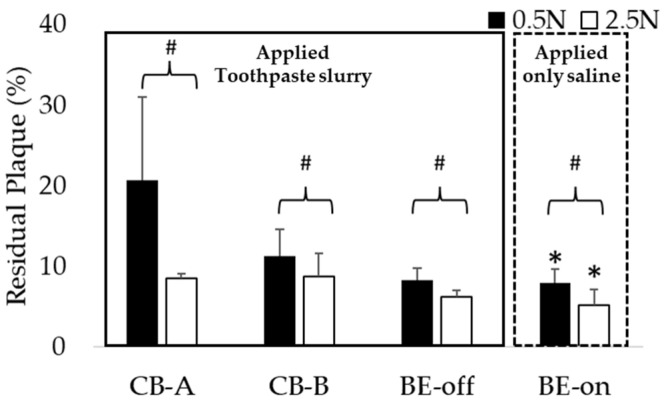
Comparison of residual plaque in applied toothpaste slurry with a typical toothbrush versus applying only saline with BE-on. Results are presented as means ± SDs. * *p* < 0.05 versus CB-A, # *p* < 0.05 versus 0.5 N.

Through this comparison, we investigated whether BE could replace toothpaste. We investigated whether using BE-on with only saline resulted in significantly lower residual plaque than using other toothbrushes with toothpaste and saline slurry under all pressure conditions. As shown in Figure 8, the highest plaque removal efficiency was observed for BE-on without toothpaste at 2.5 N. These results suggest that applying BE to a toothbrush can replace toothpaste.

**Figure 9:**
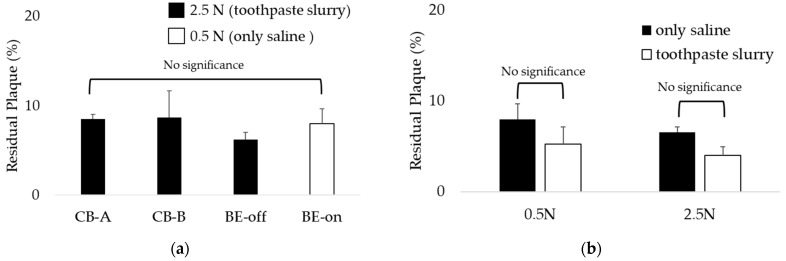
The dependence of BE on toothpaste: (**a**) Comparison of BE-on at 0.5 N in only saline versus other toothbrushes at 2.5 N in a toothpaste slurry; (**b**) comparison of BE-on using only saline versus a toothpaste and saline slurry. Results are presented as means ± SDs. There was no significant difference in residual plaque.

Interestingly, the BE-on under the 0.5 N condition using only saline showed residual plaque levels similar to those using other toothbrushes under the 2.5 N condition using toothpaste and saline slurry, as shown in Figure 9a. This result suggests that even if brushing is weak without toothpaste, plaque can be removed similarly to general brushing conditions if BE is applied. Also, as a result of comparing the residual plaque of BE-on using only saline or a toothpaste and saline slurry, there was no significant difference, as shown in Figure 9b. Therefore, if BE is applied, the dependence on toothpaste for plaque removal is expected to decrease.

## 4. Discussion

In both the only-saline and toothpaste-slurry experiments, there was no significant difference in residual plaque among toothbrushes except for BE-on. According to a previous report, there was no significant difference in plaque removal efficiency according to toothbrush bristles alone [53], similar to the results of the present study. There is a report showing that brushing under high-pressure conditions is more effective in removing plaque than under low-pressure conditions [54]. Similar results were observed in this study as residual plaque was lower under high-pressure (2.5 N) conditions. The use of toothpaste also affected the plaque removal efficiency [16,17,18]. The condition of using toothpaste was 34.67 ± 33.10% more effective for plaque removal on average than not using toothpaste. This result is similar to that of a previously reported study [55].

BE-on had a significantly lower residual plaque than other toothbrushes without BE under the same condition (Figure 6 and Figure 7). Plaque is a type of biofilm. Previous studies have reported that the treatment efficiency of biofilm is high when BE is applied [29,30,31]. BE is caused by the propagation of electromagnetic currents, waves, and voltages. Utilizing an AC at a specific frequency can enhance the porosity of the biofilm’s structure by inducing vibrations [35,36]. Conversely, a DC can induce changes in the electrolyte state [31,37]. The experimental condition is an electrically dielectric state due to only saline or toothpaste slurry being used. Saline has been used in several studies to simulate saliva [51,52]. It seems that effective propagation of the electric field can increase the biofilm removal effect. Therefore, a toothbrush with BE is expected to have a higher plaque removal efficiency.

Interestingly, it was observed that the residual plaque level using BE-on without toothpaste was significantly lower than that using toothbrushes with toothpaste under the same conditions, as shown in Figure 8. Because the BE weakens the structure of biofilm [31,32,33,34], it can increase biofilm removal efficiency. Based on these results, we propose BE as a possible substitute for toothpaste. Regardless of toothpaste application, no significant difference was observed in the residual plaque of BE-on (Figure 9b). This result indicates that if BE is applied, the dependence on toothpaste for tooth brushing can be reduced. These results mean that BE can increase the plaque removal rate regardless of the presence or absence of toothpaste. There is controversy regarding the safety of toothpaste due to abrasives, surfactants, and fluoride [16,17,18]. Generally, it includes potential carcinogenic risk, skeletal deformity, and gastric mucosal damage [18,19,20]. In children, infants and fetuses, this includes developmental disorders and bone un-development [17,22,23]. However, BE applies a microcurrent of 40.7 ± 1.5 μA, so it is harmless to the human body [46]. Thus, we anticipate that BE will be a good option for people who find it difficult to use toothpaste because of its controversial features.

Studies have reported that brushing teeth under high-pressure conditions can accelerate tooth wear [42,43]. In this study, we found that there was no significant difference in residual plaque between BE-on at a low pressure (0.5 N) without toothpaste and other toothbrushes at a high pressure (2.5 N) and with toothpaste (Figure 9a). Under the condition of the lowest pressure, brushing with only BE applied without toothpaste showed a plaque removal efficiency similar to that using the highest pressure and toothpaste. This means that a toothbrush using BE can show good plaque removal efficiency without excessive brushing or toothpaste. Therefore, it can be proposed that the application of BE can effectively remove plaque even without toothpaste or aggressive brushing. Hard brushing can increase tooth wear [42,43]. Thus, this suggests that the application of BE may reduce the intensity of tooth brushing. Therefore, it is expected that the wear of permanent teeth can be reduced if BE is applied. We believe that the application of BE will reduce not only the potential risk of toothpaste, but also the potential risk of tooth wear caused by strong brushing.

The use of an in vitro simulator not only has high experimental reproducibility compared to clinical trials, but it also enables quantitative testing [38]. In this study, we quantitatively compared the plaque removal efficiency of BE toothbrushes and other toothbrushes using an in vitro simulator. In our previous clinical study, a BE-applied toothbrush significantly reduced the gingival index, but not the plaque index [8]. This might be because there was variation among the subjects in the clinical study. It is possible that a significant plaque index was not observed due to human bias. However, since in this study we conducted an in vitro experiment using a brushing simulator capable of quantitative evaluation, we were able to observe significant plaque removal efficiency.

Based on these research results, we intend to additionally develop various oral care products including toothbrushes. Plaque can cause various problems in the oral cavity; not only on the teeth, but also on parts such as the tongue [56]. This applies not only to people; pets also have various problems due to plaque [57]. Therefore, we plan to develop oral care products through mechanical improvement with BE, including tongue cleaners and pet toothbrushes, to solve various problems related to plaque.

In this study, we evaluated the plaque removal efficiency of a BE toothbrush in a simulated brushing environment. However, our study has some limitations. First, we used consistent brushing speed and pressure, which might not reflect the variability in brushing patterns that occurs in real life [58]. Second, we used the same typodont for our tests, which does not account for differences in oral cavity structure that exist from person to person [59]. Third, we only evaluated plaque removal in one area of the teeth, although the structure of teeth varies from region to region. There is a possibility that the plaque removal rate may vary depending on the brushing pattern, tooth structure, and area. Therefore, additional studies are needed to determine the plaque removal efficiency of BE in more realistic conditions. These studies should investigate the effects of different brushing patterns, pressure conditions, and oral cavity structures.

## 5. Conclusions

In this study, we developed a BE toothbrush and investigated the plaque removal effect of BE through quantitative plaque removal experiments using a brushing simulator. A BE toothbrush was observed to be effective in removing plaque. We believe that the application of BE will contribute to effective oral care. In addition, the application of BE not only shows the possibility of replacing toothpaste, but also shows the ability to reduce the intensity of brushing. Thus, it is expected that the wear of permanent teeth can also be reduced. In the future, we will develop various oral care products applying BE, including tongue scrapers, toothbrushes for pets, and so on.

## Figures and Tables

**Table 1:** Description of condition for tested toothbrushes.

Abbreviations	Toothbrushes	BE Applied
CB-A	2080 Original Toothbrush, Aekyung Co., Ltd., Seoul, Republic of Korea	X
CB-B	Oral-B Ultra-fine, Oral-B Laboratories, Boston, MA, USA	X
BE-Off	Non-bioelectric effect	X
BE-On	0.7 V amplitude of 10 MHz with 0.7 V offset	O

**Table 2:** Details of bioelectric signals [8,45].

Contents	Details	Comments
Intensity	0.7 V	Below-water electrolysis 0.82 V
Frequency	10 MHz	Effective frequency
Composition (AC:DC)	1:1	Effective biofilm treatment

## Data Availability

Not applicable.
